# The Welfare of Pig-Hunting Dogs in Australia

**DOI:** 10.3390/ani9100853

**Published:** 2019-10-22

**Authors:** Bronwyn Orr, Richard Malik, Jacqui Norris, Mark Westman

**Affiliations:** 1 Sydney School of Veterinary Science, The University of Sydney, Sydney 2006, Australia; jacqui.norris@sydney.edu.au (J.N.); mark.westman@sydney.edu.au (M.W.); 2 Centre for Veterinary Education, The University of Sydney, Sydney 2006, Australia; richard.malik@sydney.edu.au

**Keywords:** dog, hunting, feral pig, welfare, Australia

## Abstract

**Simple Summary:**

The hunting of feral pigs utilising dogs is a common recreational activity in Australia. These dogs assist hunters to find, pursue, and restrain feral pigs. It is a legal activity in some states and territories of Australia while it is banned in others and is considered a controversial method of pest control. Scant information is available in the peer-reviewed literature regarding the welfare of dogs used in pig hunting. We conducted a review of the limited scientific literature on working dogs in order to determine the health and welfare risks that pig-hunting dogs might face. Possible risks identified include breeding surplus to requirements, the early retirement of dogs due to behavioural incompatibilities, use of punishment-based training techniques including electric shock collars, keeping dogs isolated in kennels or on tethers, exposure to numerous infectious diseases, high rates of traumatic injuries, poor transportation methods, high mortality during hunts, and suboptimal quality of life after retiring from hunting. There are also concerns about the welfare of the wild pigs being hunted by the dogs. We conclude that more research is required into the health and welfare of pig-hunting dogs. The humaneness of this method of pest control urgently requires further assessment.

**Abstract:**

Hunting feral pigs using dogs is a popular recreational activity in Australia. Dogs are used to flush, chase, bail, and hold feral pigs, and their use for these activities is legal in some states and territories and illegal in others. However, there is little knowledge about the health and welfare of dogs owned specifically for the purpose of pig hunting. We conducted a review of the literature on working dogs in Australia and overseas to determine the likely welfare impacts confronting pig-hunting dogs. We identified numerous challenges facing pig-hunting dogs throughout their lives. Risks to welfare include overbreeding, wastage due to behavioural incompatibilities, the use of aversive training techniques including electronic shock collars, solitary kenneling and tethering, high exposure to infectious diseases including zoonotic diseases, inadequate vaccination and anthelmintic prophlyaxis, high incidence of traumatic and other injuries during hunts, climatic exposure during transportation, mortality during hunts, and a suboptimal quality of life after retirement. There are also significant welfare concerns for the wild pigs hunted in this manner. We conclude that research needs to be conducted in order to determine the current health and welfare of pig-hunting dogs, specifically in Australia. The humaneness of this method of pest control urgently requires further assessment.

## 1. Introduction

The use of dogs (*Canis lupus familiaris*) as aides for hunting has a long history. With domestication beginning 15,000–33,000 years ago [1], they are featured on carvings in the Arabian Peninsula in Western Asia that are more than 8000 years old. These carvings show leashed dogs being used by humans to hunt deer, the first evidence that humans used dogs as a hunting aide [2]. In Australia, dogs are commonly used to hunt feral pigs (*Sus scrofa*) for pest management as well as recreational and commercial reasons [3]. Feral pigs quickly spread across the Australian landmass following their introduction to New South Wales (NSW) in 1788 by European settlers [4] and are now estimated to have spread to more than 45% of the continent [3]. There were an estimated 13 to 23 million feral pigs in Australia in 1990, although pig numbers are thought to fluctuate widely according to climatic conditions [5]. Considered as one of Australia’s most invasive and damaging pest animals [3], feral pigs are a ‘declared pest animal’ in all states and territories, placing an onus on landholders to actively control their numbers [6,7,8]. 

Feral pigs in Australia are managed through a variety of control methods, including poisoning (commonly using fluoroacetate [compound 1080] laden grains as bait), aerial and ground shooting, trapping, as well as recreational and commercial hunting [3,9]. While poisoning is the most common and effective control method employed by private landholders, municipal councils and state governments, hunting is also widely encouraged and practiced as a control method [3,10,11]. There is a strong hunting culture in rural Australia, backed by the political group The Shooters, Fishers and Farmers Party who often hold the balance of power in state and federal conservative governments. Along with the absence of a national, evidence-based control program for feral pigs, pig hunting remains a popular control method despite evidence calling into question its effectiveness [3,4,6,9]. In areas with the appropriate infrastructure, such as chiller boxes, commercial hunting of feral pigs is undertaken primarily for the wild boar export meat market [3]. Commercial wild pig hunting in Australia is a small and unpredictable industry, with recreational hunting far exceeding the commercial industry in terms of number of participants, number of pigs harvested and land coverage [3,11]. 

Recreational hunters are overwhelmingly male (98%) according to a recent survey of recreational hunters in Australia. The almost exclusively male participants reinforce the masculinity associated with hunting. The number of recreational hunters in Australia have been estimated at between 200,000 and 300,000 individuals (0.8%–1.2% of the national population) [11]. Although the exact number of hunters in Australia who target feral pigs is not known, based on the prevalence of the activity in hunting magazines and responses to previous hunting participation surveys, it is likely that feral pig hunting is one of the most popular hunting activities in Australia [3]. In a survey of pig hunters in 2016, 52% used dogs as a hunting aide, with one or two dogs being used per hunter [10]. It is suspected that hunters don’t use all their dogs for every hunt, as a New Zealand (NZ) study found pig hunters generally owned three dogs each, with some owning up to eight dogs for hunting purposes [12]. 

Hunting feral pigs with dogs is popular in many countries, particularly in NZ, the United States of America (USA), France, Belgium, and Greece [12,13,14]. Dogs are also used to hunt other species overseas including deer, foxes and hares [15,16,17,18,19,20,21]. Although hunting with dogs has a long history in many cultures, it is not without controversy. The United Kingdom (UK) banned hunting with dogs in 2004 after widespread public outcry and a government inquiry that found severe animal welfare risks associated with the practice [16]. The role of hunting dogs in spreading infectious diseases through livestock and wildlife populations in the USA and UK has been highlighted recently, further fueling concerns about hunting practices in these countries [13,22]. Welfare concerns have also been raised about both the welfare of quarry species pursued by dogs [15,18,23,24,25,26,27,28,29,30] and the dogs themselves [15,16,23,25,26,27,29,30,31,32,33]. 

Although the activity of hunting introduced animals in Australia is generally supported by municipal councils [34,35] and state governments [36] through the provision of bounties and access to state-owned land for hunting, support of councils and governments for pig-hunting dogs themselves is less forthcoming. Some jurisdictions, such as the state of NSW, have declared hunting dogs as ‘dangerous’ [37,38], making them subject to additional movement and housing restrictions. Other jurisdictions such as the Australian Capital Territory (ACT) have used pig hunting as a reason for euthanasia during a dog attack case, citing concerns about the risks to public safety posed by dogs who have participated in pig hunting [39,40]. Every state and territory in Australia has different laws concerning dogs used for hunting (Table 1). 

In states such as NSW, the division of legislation between municipal councils and state government departments has created a confusing legislative environment for owners of pig-hunting dogs. Under the NSW Companion Animals Act (1998), pig-hunting dogs are a declared dangerous dog [37]. Additionally, the municipal council of the Upper Hunter Shire in NSW (approximately 250 km north of Sydney) has considered introducing by-laws which would restrict pig-hunting dog movement in populated areas [41]. In contrast, other NSW government departments actively support hunting feral pigs with dogs [36] and NSW has some of the most generous animal welfare exemptions for pig hunting in the country, allowing dogs to bite and hold (or ‘lug’) onto pigs during hunts [42].

There is a lack of a basic evidence concerning the health and welfare of dogs used to hunt feral pigs and no assessment has been performed on the humaneness of using dogs to hunt pigs. Indeed, the past two reviews of federal government initiatives into the humaneness of current feral pig control methods failed to qualitatively assess pig hunting with dogs due to a lack of information [28,29]. As hunting is often purported by supporters as an effective pig control method [43,44,45], despite evidence to the contrary [9,24,26,28,29,46], this is a topic for further research. 

This review explores the welfare of pig-hunting dogs in Australia by drawing on the international literature, working-dog research and available local research. We used the classical definition of animal welfare by Donald Broom, that is “the welfare of an individual is its state as regards its attempts to cope with its environment” [47]. Further, we considered the three primary domains favoured by David Fraser as factors that impacted animal welfare, including physical health, affective states, and ‘telos’, or naturalness [48]. Using these definitions and parameters, we explored the whole-of-life experience of pig-hunting dogs, from breeding and rearing, through to housing, health, nutrition, and hunting activities, to finally consider the impacts of retirement. 

## 2. Early Life

### 2.1. Breeding 

**Figure 1:**
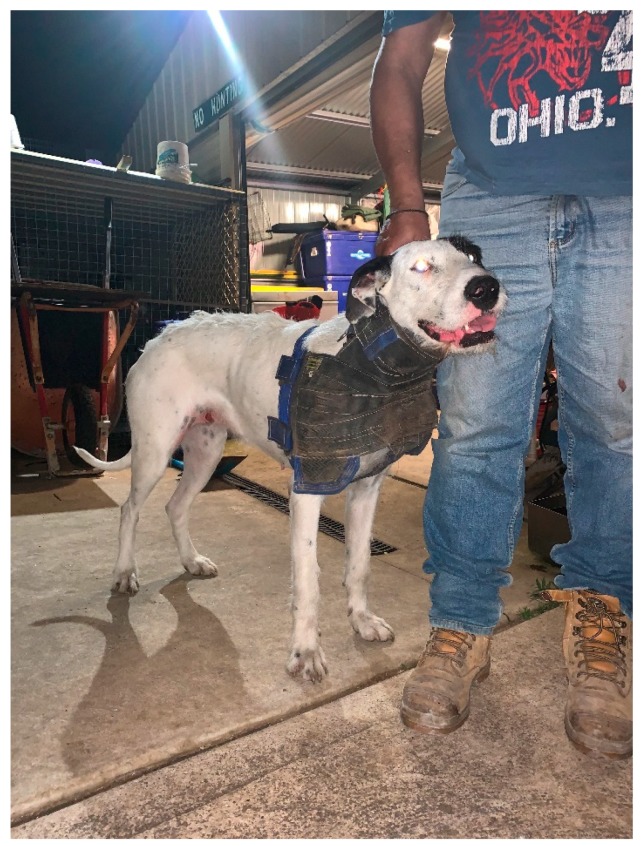
Bull Arab pig-hunting dog in protective armour in Australia (*author supplied*).

Most pig-hunting dogs in Australia are a mixture of various pedigree breeds [25]. They generally have medium to large builds, with composite types such as the Bull Arab (Figure 1) used to hunt large boars [49]. Pig-hunting dogs in Australia, including the Bull Arab, are a mixture of ‘bully breeds’ such as Bull Terriers, Bullmastiffs, Staffordshire Bull Terriers and American Pit Bull Terriers, as well as leaner, sight hound breeds such as Greyhounds, Wolfhounds and Staghounds (colloquially known as ‘hairy dogs’), German Shorthaired Pointers and Catahoula Leopard Dogs [50]. This breed composition differs from nearby NZ where, according to one study, Cattle Dogs were the most popular breed, followed by Greyhounds and Bull Terriers [12]. Hunting requires dogs to expend energy in short, intense bursts during chases and in longer, sustained periods when searching for quarry in dense vegetation, similar to the activity patterns of working sheep dogs [51,52]. Sires and dams are often chosen based on their hunting ability, boldness, intelligence, and strength [53]. As most pig-hunting dogs are sexually entire [12], unintended mating during hunts and subsequent accidental litters are possible.

It is currently unknown how many pig-hunting dogs are bred in Australia annually, however an estimate can be derived from the literature. In 1982, it was estimated that between 100,000 and 200,000 people engaged in pig hunting in Australia [14], and in 2016 it was determined that 52% of pig hunters in Australia use dogs [10]. As per above conservative estimates, 52,000 pig hunters in Australia own dogs. A survey of working dogs in 2009 revealed an average of 15 puppies born annually in working dog owning homes which included hunting dogs [54]. A recent NZ study found pig hunters owned on average three adult hunting dogs, with some hunters owning up to eight dogs [12]. This means there are more than 156,000 adult pig hunting dogs in Australia, at an average of three adult dogs per hunter, with 15 puppies born per annum, if fertility rate estimates are considered similar to other working dogs. Going by these estimates, which exclude infant mortality, neutered dogs and breeding not done by all owners, 780,000 pig-hunting dog puppies might be born annually in Australia. 

Although the extent to which pig-hunting dogs are overbred is unknown, it is likely that, with a large population of entire animals, some dogs are bred as surplus for replacement requirements. Indeed, overbreeding and high numbers of culled animals are a problem across Australia’s working dog industries, with significant ‘wastage’ occurring in young animals [54]. The primary driver of this wastage is behavioural incompatibility [55,56] including a lack of drive, although most of these issues aren’t identified until dogs are adults, meaning the breeding and culling of young adult working dogs is a welfare concern. 

It is unknown if pig-hunting dogs encounter reproductive issues such as infertility and dystocia. Infertility has a variety of causes including infectious diseases such as brucellosis. Brucellosis in Australian dogs is caused by *Brucella suis* and has been frequently reported in pig-hunting dogs in NSW [57] and Queensland (*Dr Cathy Kneipp*, *pers. comm*.). Clinical signs of brucellosis include an undulating fever, discospondylitis, and reproductive disease due to a predilection for testicular and ovarian tissue, causing infertility, abortions and stillbirths in dogs [58]. It is likely that some pig-hunting dogs experience reproductive issues caused by *B. suis* infection.

As they are bred for purpose, rather than for somatotype, pig-hunting dogs probably experience lower dystocia rates than purebred ‘bully breed’ dogs such as the British Bulldog which struggles with more extreme brachycephalic confirmation [59]. 

### 2.2. Training and Induction

A dog’s personality is affected by both genetic and environmental factors. Although genetics play a key role, with studies clearly showing the personality of the parents impacts offspring temperament [60], environmental inputs also play a formative role [61]. The peak socialisation period for all puppies is between 3 and 12 weeks-of-age [62]. During this time, dogs learn about conspecifics and become desensitised to environmental stimuli. A deprived socialisation period, such as prolonged periods in a barren kennel, will result in puppies with heightened anxiety, fear and apprehension towards novel stimuli [63,64,65]. It is unknown what socialising, if any, pig-hunting puppies currently receive. It has been reported that pig-hunting dogs are very ‘stoic’ and safe around children [45,49], but there have also been reports of pig-hunting dogs attacking humans [38] and other dogs [39,40]. It is likely, given the diverse nature of the activity, that the socialisation of pig-hunting dogs varies widely. 

There is some information available on the methods used to train pig-hunting dogs. A survey of working dog owners, which included hunters, found the use of electronic shock collars and other aversive training techniques was common among handlers who hadn’t received any formal dog training qualifications [54]. Anecdotally, several social media groups frequented by pig hunters such as ‘Pig Dogging and Hunting Items for Sale—Qld and Beyond’ and ‘Pig Hunting Gear Dogs Utes Equipment for Sale’ advertise electronic shock collars for purchase. Discussion on these forums includes the best types of collars as well as techniques for their use in training, such as preventing dogs from attacking non-target animals like livestock. Contrarily, research shows that electronic shock collars are detrimental to dog learning [66,67] and owners using them in working dogs are less likely to have successful dogs [56]. Electronic shock collars and other aversive training techniques rely on the use of punishment and fear to curb behaviour and are frequently criticised for their adverse impact on dog welfare, including increasing anxiety and aggression as well as reducing motivation [68]. 

## 3. Adult Life 

### 3.1. Housing and Enrichment

The housing environment of pig-hunting dogs in Australia likely varies according to their perceived role (i.e., companion vs working dog), the commitment of their owner to the activity (amateur vs professional hunter) and the number of dogs kept on the property. Single kennels are the most common form of housing for pig-hunting dogs in Australia [44]. Other types of housing include single tethering [69] and living in residential backyards [45]. In some states of Australia, codes of practice exist that outline how working dogs should be kept [31,69,70], although these are just recommendations and are not legally enforceable.

The impact on dogs being housed individually in kennels has been studied extensively [56,71,72,73,74,75,76,77,78]. While many studies report dogs housed in single kennels experience high levels of stress and frustration [56,72,73,74,76,77], others have found no impact of long term kenneling on cortisol levels and behaviour, although tools for measuring affective states may limit the ability to measure this impact [71,75]. Many authors have proposed that a dog’s ability to adapt to kenneling varies on an individual basis [71,76,77,78]. Barren kennels, with an absence of bedding, toys, and other enrichment items, pose welfare challenges [73,74,79]. It is currently unknown whether pig-hunting dogs kept in kennels have access to bedding or enrichment items, although research into other working dogs in Australia has found that most dogs are kept in barren kennels [56,72]. Most states and territories in Australia have laws which require dogs to be provided with minimum time outside confinement, the general recommendation for which is to provide 30 min to 2 h outside the kennel every 24 h [80]. Given the rural and remote locations in which many kenneled dogs in Australia live, it is unlikely these laws are enforced.

Tethering dogs also presents potential welfare challenges, as it can expose dogs to extreme weather if no shelter is provided and allows little environmental comfort [81]. There are reports of deaths of tethered dogs due to tipping of water bowls [82] or when these were out of reach [83]. Pig-hunting dogs kept in kennels are also at risk of dehydration or starvation due to their strict confinement if owners fail to attend the kennels [84]. 

### 3.2. Health and Nutrition

To hunt effectively, pig-hunting dogs need to be in optimal health. Running upwards of 40 km a day means that many pig-hunting dogs have lean musculature [44,45,69], and rarely encounter health conditions such as obesity that are common in neutered pet dogs [85]. Nutrition and weight management can, however, still be an issue for these athletic dogs. Due to the high energy expenditure of hunting dogs [51], care must be taken to avoid dogs becoming underweight [27]. Although most pig hunters feed their dogs commercial diets [12], not all such diets are suitable for hunting dogs [86]. In addition, many hunters choose to supplement commercial diets with, or feed entirely, raw game such as feral pig and kangaroo [12,31]. Feeding raw offal and muscle to dogs from hunted wild animals or fallen stock has potential health risks. In the UK, a group of Fox Hounds contracted *Mycobacterium bovis* (causative agent of bovine tuberculosis) after being fed raw meat [87]. In NZ, an idiopathic myopathy in dogs known as ‘Go Slow’ has been linked to the feeding of raw feral pig [12]. In Australia, dogs fed raw feral pig are potentially exposed to echinococcosis caused by tapeworm *Echinococcus granulosus* [88], sparganosis caused by *Spirometra erinaceieuropaei* [89], and bacterial agents of gastrointestinal disease including *Salmonella* spp. and *Escherichia coli.* [90]. Pig-hunting dogs can also be exposed to a wide range of infectious agents during hunts from either the environment or feral pigs including *Coxiella burnetii* (the causative agent of Q Fever) [91], *Burkholderia pseudomallei* (the causative agent of melioidosis) [4], various *Leptospira* spp. and *Brucella suis* [92]. Most of these diseases are zoonotic, with hunters also potentially exposed through contact with either feral pigs or their dogs.

Preventative veterinary medicine practices in Australian pig-hunting dogs are currently unknown. In a NZ study on pig hunters, it was reported that 58% (114/194) of pig-hunting dogs had seen a veterinarian in the preceding 12 months, although most of these visits were for wound management [12]. A 2018 study on NZ farmers found 40% (69/171) of farm dogs were infected with gastrointestinal parasites, despite anthelmintic treatments [93]. Prophylaxis against intestinal worms, heartworm, fleas, and ticks in pig-hunting dogs is not known [10]. Microchipping of pig-hunting dogs or registration with respective local councils is also unknown, although both identification methods are legal requirements in many parts of Australia. Several common diseases, such as canine parvovirus and certain strains of leptospirosis, are preventable through vaccination, although there are no data available regarding how many pig-hunting dogs are vaccinated against these diseases. Most pig-hunting dogs are entire [12], which is a trend replicated in the wider working dog population [55]. There is a widely-held belief that neutering working animals decreases their performance, despite research showing no compromise to working ability when chemically castrated [94]. 

The greatest risk to a pig-hunting dog’s health is from the hunt itself. Although hunting dogs likely experience positive affective states during hunting as a result of their strong prey drives [19], they are at increased risk of heat exhaustion, poisoning, vehicular trauma, snake bite, accidental shooting and dehydration while hunting [25,29,30,31]. Additionally, feral pigs often have sizeable tusks that can penetrate a dog’s chest or abdomen during an encounter [44]. Although many hunters now use armour such as chest plates made from leather (Figure 1), the efficacy of these protective devices is unknown, and many injuries are still reported by veterinarians who treat these dogs [23,25,26]. Well-stocked first aid kits with antibiotics, staple guns, and trauma kits often accompany hunters during outings, resulting in delayed or absent veterinary attention for some dogs [12,26]. The welfare implications associated with poorly or untreated traumatic wounds are severe, with pain, sepsis and eventual death potential outcomes. 

## 4. Hunting Related Activities

### 4.1. Transportation

Transport to and from hunting locations often requires travelling significant distances in the rural countryside. Dogs are generally transported in metal cages located on the tray of utility vehicles (‘utes’) and are frequently seen accompanying their owners on errands in country towns [41]. Pig-hunting dogs are often left in these cages whilst owners conduct their business, with most cages an open wire mesh design providing minimal shade [25]. Multiple dogs are transported simultaneously in these cages, with no mandated minimum space requirements for transporting dogs in Australia, although research has shown that dogs experience greater comfort during car travel when afforded more space [95]. The biggest welfare risks associated with this form of travel are heat exhaustion and dehydration due to a lack of shade and the hot conductive surfaces of metal ute trays [96]. 

### 4.2. Hunting Expeditions 

During pig hunting expeditions, dogs may get injured or lost [10]. If lost, dogs are susceptible to dehydration, starvation or joining the feral dog population [25,27]. Many hunters now use GPS collars and strobe lights to reduce the number of dogs that go missing [10]. 

According to a recent survey of recreational hunters in Australia, most use guns to kill prey animals [11]. However, 16% do not use guns, and rely on knives or arrows. Sampling bias favouring hunters who use guns was observed in this survey, as it was publicised by the Sporting Shooters Association, with other publications reporting Australian hunters’ preference for dogs and knives to kill feral pigs [3,97]. One reason speculated for this preference is, in part, because of Australia’s strict gun laws [97], which makes acquiring a firearm as a recreational hunter more difficult than in countries like the USA. 

Although little published information exists detailing the specifics of pig hunting expeditions, we can form an outline of pig hunting activities by using available studies, media reports, and research from welfare organisations. There are two main hunting methods used with dogs: (i) finding and flushing pigs into open areas to be shot using rifles or arrows and (ii) finding, bailing, holding, or lugging pigs so a hunter can dispatch the pig with a knife [29]. 

Using a knife to kill an unrestrained pig can be dangerous to both the hunter and the dog. In this method of hunting, dogs are required to chase, find, bail, and then lug or hold onto a pig for restraint. Generally, two or three dogs bite and hold onto a pig, with two on each ear and sometimes a third dog biting a leg or other extremity [29]. Biting the ear of a boar that possesses large, sharp tusks is risky, and a dog that hesitates or loses its grip may be slashed or stabbed with a tusk. Once a pig is ‘restrained’ by the dogs, a hunter will generally come up behind it and lift a rear leg, to allow safe access to the chest and abdomen. A large knife is then used to stab the pig, with most hunters aiming for the heart, lungs, and great vessels. Once the pig has been stabbed several times, the hunter usually releases the leg and retreats, waiting for the pig to collapse [26,29,44]. Often hunters will recall their dogs away from the pig at this stage, although as video footage on social media sites such as YouTube show, many hunters do not [23,26]. 

### 4.3. Pig Welfare

This paper is primarily concerned with the welfare of dogs used to hunt feral pigs, but it would be remiss not mention the welfare impacts on the pigs themselves. Hunting pigs with dogs likely elicits a similar physiological reaction to that found in deer hunted by dogs [18]. Although the chase during feral pig hunts is shorter than in deer hunts, the intensity of the fear and stress experienced by the pig is likely comparable [29]. In addition, hunting pigs with dogs and knives results in pain and trauma when the dogs bite the pig, with the amount of time this restraining period occupies during a hunt currently unknown. Once stabbed with a knife during the dispatch phase of the hunt, the length of time to death could take anywhere from seconds to minutes depending upon what organs are lacerated. Although stunning prior to death is a common practice in Australian abattoirs, hunted feral pigs are conscious during exsanguination [26,28,29]. Conscious exsanguination is known to be painful, distressing and result in severe welfare compromise [98]. It is also unlikely that hunters verify a pig is dead by checking reflexes such as the corneal reflex or rhythmic breathing as per commercial abattoir requirements [99]. The method of using dogs and knives to kill feral pigs, therefore, undeniably results in substantial pain and suffering to the pig.

## 5. Later Life

### 5.1. Retirement

The retirement of working dogs is a contentious subject [56,72]. Scant longitudinal data exists on the retirement outcomes of working dogs and working dogs may retire early into their careers due to injuries [100], poor performance or behavioural problems [55,56,72]. Dogs that leave working employment can follow several pathways, including being given away or sold as a working dog, breeding stock or pet, being euthanased by the owner or by a veterinarian, or being kept by the owner as a pet or breeding stock [54,55,56,73]. There is currently no data concerning the outcomes of pig-hunting dogs following retirement. The average age of retirement for Australian farm dogs was found to be 10 years [55], but it is unknown at what age pig-hunting dogs are retired. A NZ study reported that the average age of active hunting dogs was three years [12], suggesting a disproportionate number of younger dogs in work. Retired working dogs can also have difficulties transitioning to life as companion animals, with potential hurdles including housetraining, unfamiliar environments and routines and reduced exercise [100]. Pig-hunting dogs, with their high prey drives and kenneled lifestyle, may face problems in retirement similar to ex-racing greyhounds [101]. 

### 5.2. Death and Destruction Techniques 

Many owned dogs have their lives actively terminated, generally through veterinary euthanasia [102]. Working dogs are no exception, and a survey into the outcomes of farm dogs in Australia found 21% were euthanased upon retirement [55]. Veterinary euthanasia of dogs is considered widely acceptable as a humane procedure, with minimal welfare compromise involved [103]. It is legal for farmers and working dog owners in Australia to destroy their dogs humanely when they are no longer wanted, with most using a single gunshot to the head [31]. This can be a humane euthanasia method if done correctly. 

Working dogs can also die during the course of their work [29,32,55,103]. Approximately 31% of Australian farm dogs in one survey died during their working career [55], with death on duty a hazard for police and military dogs as well [104]. Pig-hunting dogs encounter many risks during their hunting expeditions, including heat exhaustion, poisoning, accidental shooting, traumatic injuries, snake bites, and vehicular accidents. Considering the many risks facing these dogs, death during or as a result of hunting expeditions is probably not an uncommon ending to a pig-hunting dog’s life [26,28]. 

## 6. Conclusions

Although conducting a review of the literature is a useful method to gain context and perspective, it has several major limitations. In the absence of our own data, we extrapolated findings from similar situations overseas and with Australian farm dogs and based many of our findings on assumptions of current practice. It is important to further study the health and welfare risks faced by pig-hunting dogs in Australia. As hunting pigs with dogs occurs out of view of the public, often at night and in rural areas using unregistered dogs, pig hunting is a practice that has largely escaped formal study and scrutiny. Future research needs to address the husbandry and working of pig-hunting dogs in Australia. A humaneness assessment of the use of these dogs for pest control is needed. 

## Figures and Tables

**Table 1:** Legislation relating to pig-hunting dogs in Australia.

State/Territory	Animal Welfare Legislation	Domestic Animal Legislation	Is Hunting Pigs with Dogs Legal?	Additional Considerations
Queensland	Animal Care and Protection Act 2001	Animal Management (Cats and Dogs) Act 2008	Yes ^#^	Compulsory microchipping
New South Wales	Prevention of Cruelty to Animals Act 1979 Prevention of Cruelty to Animals Regulation 2012	Companion Animals Act 1998	Yes	Dogs kept for pig hunting are subject to ‘dangerous dog’ requirements such as secure housing Compulsory microchipping
Australian Capital Territory	Animal Welfare Act 1992	Domestic Animals Act 2000	No	Compulsory microchipping Non-breeding dogs must be desexed by six months of age
Victoria	Prevention of Cruelty to Animals Act 1986	Domestic Animals Act 1994	Yes *	Compulsory microchipping
Tasmania	Animal Welfare Act 1993	Dog Control Act 2000	Not applicable ^	Compulsory microchipping
South Australia	Animal Welfare Act 1985	Dog and Cat Management Act 1995	Yes *	Compulsory microchipping Must wear ID tags unless on private land
Western Australia	Animal Welfare Act 2002	Dog Act 1976	Yes	Compulsory microchipping
Northern Territory	Animal Welfare Act 1999 Animal Welfare Regulations 2000 Animal Protection Act 2018 (not yet commenced—awaiting regulations)	Council by-laws only	Yes	

^#^ Queensland legislation on allowing dogs to hold or lug feral pigs is unclear. It is an offence exemption so long as ‘unreasonable pain and suffering’ isn’t caused by the activity. * Victoria and South Australia allow dogs to flush, chase, and bail feral pigs but do not permit direct contact to occur, such as holding or lugging the pig. ^ There is no significant feral pig population on the island state of Tasmania.
